# Analysis of the Robotic-Based In Situ Bioprinting Workflow for the Regeneration of Damaged Tissues through a Case Study

**DOI:** 10.3390/bioengineering10050560

**Published:** 2023-05-08

**Authors:** Gabriele Maria Fortunato, Sofia Sigismondi, Matteo Nicoletta, Sara Condino, Nicola Montemurro, Giovanni Vozzi, Vincenzo Ferrari, Carmelo De Maria

**Affiliations:** 1 Department of Information Engineering, University of Pisa, 56126 Pisa, Italysara.condino@unipi.it (S.C.); giovanni.vozzi@unipi.it (G.V.); vincenzo.ferrari@unipi.it (V.F.); carmelo.demaria@unipi.it (C.D.M.); 2 Research Centre “E. Piaggio”, University of Pisa, 56126 Pisa, Italy; 3 EndoCAS Center for Computer-Assisted Surgery, University of Pisa, 56126 Pisa, Italy; 4 Department of Neurosurgery, Azienda Ospedaliera Universitaria Pisana, 56126 Pisa, Italy; nicola.montemurro@unipi.it

**Keywords:** robotic-based in situ bioprinting, material extrusion, cranial surgery, bone repair, non-planar slicing, path registration

## Abstract

This study aims to critically analyse the workflow of the in situ bioprinting procedure, presenting a simulated neurosurgical case study, based on a real traumatic event, for collecting quantitative data in support of this innovative approach. After a traumatic event involving the head, bone fragments may have to be removed and a replacement implant placed through a highly demanding surgical procedure in terms of surgeon dexterity. A promising alternative to the current surgical technique is the use of a robotic arm to deposit the biomaterials directly onto the damaged site of the patient following a planned curved surface, which can be designed pre-operatively. Here we achieved an accurate planning-patient registration through pre-operative fiducial markers positioned around the surgical area, reconstructed starting from computed tomography images. Exploiting the availability of multiple degrees of freedom for the regeneration of complex and also overhanging parts typical of anatomical defects, in this work the robotic platform IMAGObot was used to regenerate a cranial defect on a patient-specific phantom. The in situ bioprinting process was then successfully performed showing the great potential of this innovative technology in the field of cranial surgery. In particular, the accuracy of the deposition process was quantified, as well as the duration of the whole procedure was compared to a standard surgical practice. Further investigations include a biological characterisation over time of the printed construct as well as an in vitro and in vivo analysis of the proposed approach, to better analyse the biomaterial performances in terms of osteo-integration with the native tissue.

## 1. Introduction

Bone repair is a biological process consisting of the self-regeneration of the tissue that heals itself without a fibrous scar [1,2,3]. Nevertheless, bone defects with critical dimensions need to be treated with a clinical intervention [4]. Repair of bone defects is one of the challenges surgeons face in clinical practice which remains unsatisfied with traditional methods (autologous bone grafting or bone from a donor): 5% to 10% of the annual fractures fail to achieve complete repair causing harm to both the patient and the health care system [5,6]. This kind of defect needs a bone graft to fill the space, offer support and improve the defect repair capacity [7].

Considering the case of cranial bone defects, the gold standard for the treatment is autologous bone substitutes for their great properties of biocompatibility and osteointegration, despite their clinical use being restricted by donor-site morbidities and because autograft tissue is limited [8]. To avoid autograft problems, the allograft is an alternative, but it is the least used method because it is characterised by severe immunological responses, infections, bone resorption, and potential disease transmission [9,10]. Then there are synthetic materials. Metals such as titanium and its alloys are characterised by good biocompatibility but they have a high complication rate including infection, seroma, haematoma, and bleeding [11,12,13]. Furthermore, metal implants, usually produced through power bed fusion techniques, such as electron beam melting (EBM) and selective laser sintering (SLS), leads to stress-shielding effects at the implant–bone interface due to the high difference of mechanical properties with the native bone tissue [14,15]. Bio-ceramics, including hydroxyapatite, are fragile and can lead to implant fractures [16]. Poly(methyl methacrylate) (PMMA) bone cement develops heat to damage the *dura mater* and other surrounding tissue [17]. Polymeric materials such as polyetheretherketone (PEEK) have great biomechanical properties but are very expensive and do not have great osteointegration [8,18]. Moreover, all alloplastic materials bring complications including foreign body infections so the ideal material has not been found yet making it necessary to search for an alternative approach [16].

In recent years, scaffold-based solutions for cranial reconstruction have been investigated. The scaffold can be made of different biomaterials both synthetic and natural (ceramics as hydroxyapatite and calcium phosphate; metals as gold and titanium; polymers forming hydrogel as gelatin, collagen, chitosan, or thermoplastics as poly(lactic acid); composite materials combining two or more of the previously cited biomaterials) and may also include bone growth factors or stem cells [16]. The use of 3D bioprinting technology allows the scaffolds to be fabricated with the desired shape and structure [19]. In particular, the in situ bioprinting approach can lead to faster and better healing because it allows for avoiding some of the most critical procedures of the in vitro method [20]. In this way, in fact, (i) the use of a bioreactor is no longer necessary as its function is exploited by the patient’s body, (ii) the implantation procedure is not done so the risk of damage, contamination, and the problem of transportation is eliminated and (iii) the bioprinted structure perfectly matches the geometry of the damaged area [21].

The in situ bioprinting approach consists of the layer-by-layer fabrication of the tissue substitute directly on the patient’s defect site: different biomaterials or bioinks can be deposited in the damaged area to form complex architectures which attempt to replicate extracellular matrix and improve cell adhesion and their capacity to regenerate the tissue [21,22]. The two main approaches for in situ bioprinting are robotic and handheld [21]. For the case of large cranial defects, handheld bioprinting is not suitable because its success depends on human error, has a lower resolution, and lacks controlled deposition so cannot be used for complex structures [23].

In the literature, there are several attempts at using in situ bioprinting for bone tissue regeneration with different types of printers and biomaterials. For example, Keriquel et al. [24] used laser-based bioprinting (LBB) to print nanohydroxyapatite on cranial defects of mice. This study demonstrates that this approach improves bone regeneration and does not lead to deleterious defects in adjacent brain tissue. Later in 2017, they printed in situ mesenchymal stromal cells (MSC) inside a matrix of collagen and nano-hydroxyapatite comparing the impact of two different geometric arrangements of the printed cells [25]. Despite the high precision and resolution, LBB allows printing only on a flat surface and due to the low volume deposition, it takes time to reconstruct a large bone defect that limits the scope of this approach [26]. Another approach can be extrusion-based bioprinting as reported by Li et al. [8] to improve bone regeneration of long bones (e.g., femur). They used a 3D scanner to acquire the shape of the defect and successively printed a hydrogel directly on the damaged area completing the entire procedure in 10–15 min. Other researchers tried to use a handheld bioprinter with bone defects of small dimensions, allowing the surgeon to deposit the biomaterials in the site of interest. For example, Mostafavi et al. [27] used this technology to fill the bone cavity and form free-standing scaffolds of poly (caprolactone) (PCL) doped with zinc oxide nanoparticles and hydroxyapatite microparticles. The scaffold proved to support cell growth and to be well attached to the native tissue preventing temperature increase and bacterial growth. Indeed, in situ bioprinting can be used for repairing cranial defects with a critical size that represents one of the greatest challenges for surgeons, among all surgical operations. This is a clinical problem because cranial injuries happen with high frequency and are mainly due to congenital anomalies, traumas, strokes, aneurysms, and cancer. They are also one of the most widely used models to study methods for bone tissue regeneration. Generally, they need more than one surgery operation and still, traditional methods are not sufficient and fail to restore the original shape and function and therefore there is an urgent requirement for alternative treatment for this type of defect [9,26].

To date in situ bioprinting for cranial defects can represent a good method for cranial fractures. There are some critical aspects related to this procedure that are still in the research phase. Some of these defects necessitate the replacement of multiple tissue layers for the reconstruction of the tissue’s original anatomy. Moncal et al. [28] tested a hard/soft composite tissue using two different bioinks for bone and skin which include allogeneic cells and biomaterials showing that this approach can be used for large defects.

The main aspect that may or may not lead to the success of the experiment is the choice of the appropriate bioink/biomaterial ink. Biomaterials, including those mentioned above, can be printed on the defect but some researchers are looking for different solutions. The ideal bioink/biomaterial ink must be compatible, and processable with a suitable bioprinting technology and has to maintain the shape and have the right mechanical properties at physiological conditions [27,29]. For example, Moncal et al. [30] used a bioink with embedded growth factors for the reconstruction of a large calvaria defect in mice finding promising results compared to those of the empty group. In another study, vascular growth factors were instead printed together with stem cells to induce pre-vascularisation [31]. By refining the use of new bioinks and improving bioprinting technologies, better results can be achieved to revolutionise the treatment of large defect cranial bone.

In this study we present the application of in situ bioprinting to a simulated scenario, inspired by a real clinical case study, using a robotic-based in situ bioprinting approach proving the great potential of this technology. The term bioprinting is applied in this study following the definition of Moroni et al. [19], i.e., bioprinting is defined as “the use of computer-aided transfer processes for patterning and assembly of living and non-living materials with a prescribed 2D or 3D organisation to produce bio-engineered structures serving in regenerative medicine, pharmacokinetics, and basic cell biology studies”. The robotic platform IMAGObot [32] was tested to restore a cranial defect after a traumatic event onto a patient-specific phantom. A standardised workflow for robotic-based in situ bioprinting for directly regenerating a damaged area on a patient starting from medical imaging is here shown for the first time. In particular, the novelty of this work consists of the possibility of using an all-in-one platform that allows registering the planned trajectory in the operating workspace as well as depositing the desired biomaterials on the target area.

## 2. Materials and Methods

### 2.1. Robotic-Based In Situ Bioprinting Workflow

**Figure 1:**
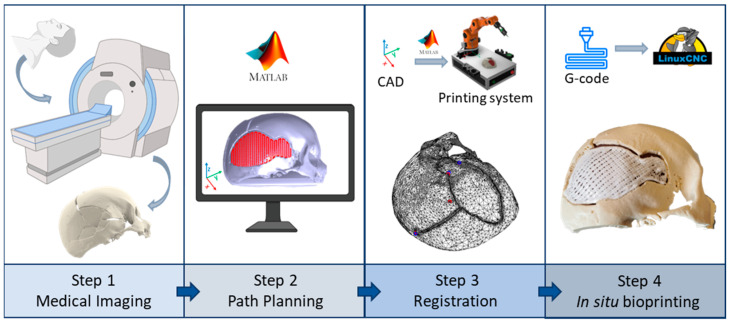
Robotic-based in situ bioprinting workflow: when the digital model of the damaged anatomical site is acquired with an external source to the robot (e.g., medical imaging), the planned path for the regeneration of the defect needs a registration step to correctly deposit the biomaterial onto the patient.

A standardised robotic-based in situ bioprinting workflow can be schematised in four main steps [33,34] (Figure 1):

1. Acquisition of the 3D digital model of the damaged area: the geometry of the anatomical portion to be reconstructed can be obtained with medical imaging techniques (e.g., computed tomography (CT), magnetic resonance imaging (MRI)) to ensure high accuracy and resolution of the model;
2. Printing path planning: based on the acquired geometry the printing pattern to restore the defect is planned. Depending on the slope of the surface on which the material has to be deposited, it is possible to use two different approaches to planning the trajectories: (i) with slopes greater than 45°, the extruder must be kept perpendicular to the surface in order to achieve an optimal deposition; (ii) on the other hand, it is sufficient to keep the extruder vertical and deposit the filler following the profile of the surface. The coordinates of the points of the planned path are referred to the computer-aided design/computed-aided manufacturing (CAD/CAM) software reference frame (e.g., in this work Matlab^®^ is used);
3. Registration of the printing path in the robot workspace: the path planning is generally defined in the pre-operative scanner reference frame, which does not coexist in space and time with the robot one, which includes the patient. The transformation matrix between the two reference frames can be computed by acquiring anatomical or artificial landmarks in both reference frames (e.g., Matlab^®^ and IMAGObot workspace);
4. Biomaterial in situ deposition: following the registration of the planned printing pattern on the patient in the operating area, using the computed transformation matrix, the in situ bioprinting process is carried out by depositing the biomaterial directly on the damaged tissue. The registered pattern is converted into a G-code and sent to the printing system control software (e.g., LinuxCNC for the IMAGObot platform).

### 2.2. Overview of the IMAGObot Platform

The experimental part was carried out using IMAGObot, a robotic biofabrication platform developed in a previous study [32,33,35]. IMAGObot is a 5 Degrees of Freedom (DoF) robotic arm designed starting from the open-source project MOVEO from BCN3D and re-engineered to be used for in situ bioprinting applications. IMAGObot is equipped with an electro-magnet as an end-effector that allows a fast and simple tool change to use different instruments during a single task [33]. In this study, a touch probe and a pneumatic syringe pump were used. The touch probe is based on a spring mechanism that, when triggered, records the spatial coordinates of the touched point. A detailed description of this probe was reported by Fortunato et al. [33], and in this new work, it is used to acquire the coordinates of fiducial markers to perform the planning registration. The syringe pump module, instead, was used for the extrusion-based bioprinting test to regenerate the cranial defect on the phantom. The robot is controlled by the open-source software LinuxCNC [36], while the path planning is carried out using the previously developed Matlab^®^ R2020a application [32,33]. This app can manage different path planning approaches starting from a generic pattern and the digital model of the printing substrate:

1. Projection method: a single layer pattern, loaded as g-code, can be projected on the substrate mesh and the end-effector path is computed constraining its orientation to be perpendicular to the printing surface;
2. Non-planar method: a single or multi-layer pattern is generated in a non-planar slicer software [37,38], uploaded in the Matlab^®^ application, and the end-effector path is computed always maintaining the vertical orientation.

IMAGObot LinuxCNC source code has been released as an open-source project on the GitHub platform (https://github.com/CentroEPiaggio/IMAGObot, accessed on 7 May 2023) and is constantly updated.

### 2.3. Case Study Implementation

#### 2.3.1. Case Study Selection and CT Scan Processing

**Figure 2:**
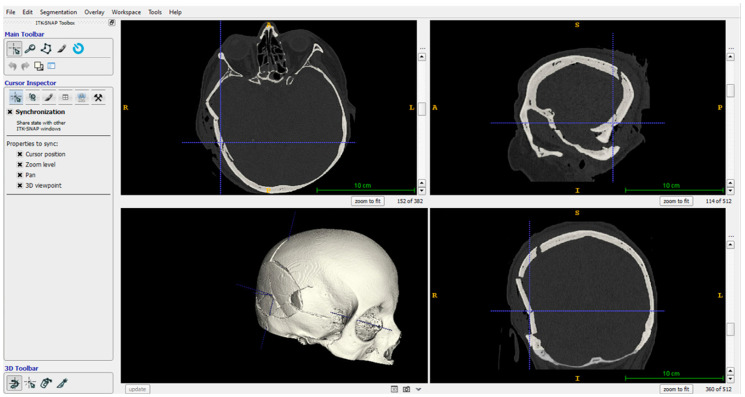
Skull anatomy segmented via the EndoCAS Segmentation Pipeline.

A skull fracture was selected as a case study to illustrate the proposed robotic-based workflow. In detail, a right fronto-temporo-parietal depressed cranial vault fracture occurred in a 68-year-old woman following high energy impact to the skull (pedestrian-car accidents) was selected. No skin wounds were present, nor underlying brain contusions. Consciousness status was quite normal, according to Glasgow Coma Scale (GCS) [39]. Indications for depressed skull fractures elevation are usually a depression > 10 mm, perhaps because of the greater likelihood of Dural tear and cerebral compression, or if there is evidence of Dural penetration, intracranial hematoma, large contusion, or focal neurologic deficit or if there is a cosmetic deformity, particularly at or near the hairline. In this last case, elevation may be needed even for a smaller depression [40]. The pre-operative CT head scan showed a right fronto-temporo-parietal depressed cranial vault fracture that met more than one of these criteria, for these reasons patient underwent surgery under general anaesthesia. A right trauma flap skin incision was performed, then all floating or depressed bone fragments were removed. No subdural hematoma was present. For this patient, the cranial vault was reconstructed by joining bone fragments with titanium plates in about 2 h and a half of surgery. The pre-operative CT head scan, featuring a 0.3×0.3×0.4 mm resolution, was processed using the EndoCAS Segmentation Pipeline integrated into the open-source software ITK-SNAP [41], to generate the digital model of the skull (Figure 2).

**Figure 3:**
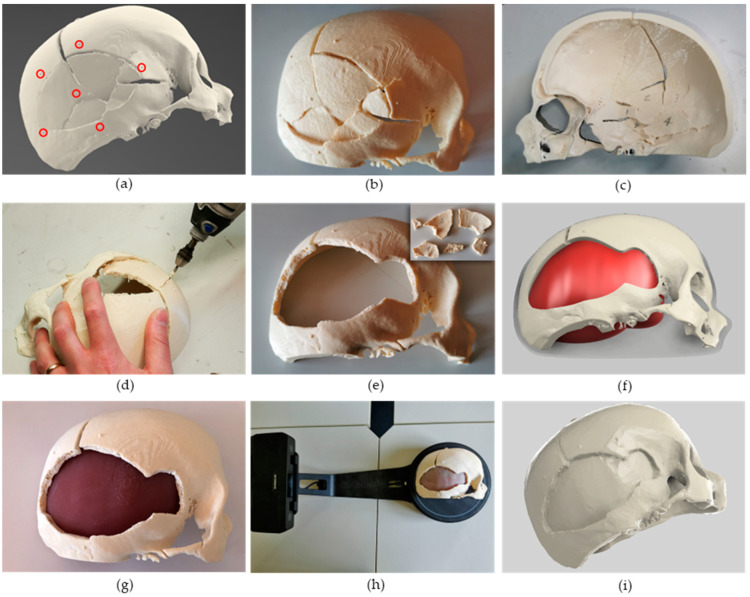
Patient-specific phantom design, fabrication, and scanning. (**a**) Digital model of the injured skull obtained through computed tomography imaging. Semi-spherical markers (2 mm diameter), highlighted with red circles, were added for the registration step. (**b**,**c**) Skull fabricated by fused-deposition-modelling technology using poly-lactic-acid. Bone fragments are both visible from the external (**b**) and internal (**b**) view. (**d**) Bone fragments were manually removed using a Dremel^®^, simulating the surgical procedure. (**e**) Obtained skull model after fragments (top right corner) removal. (**f**) Computer aided design (CAD) model of the phantom with bone and soft (*Dura Mater*) tissues. (**g**) Assembled phantom: the internal portion of the phantom was fabricated using Eco-Flex 00-10 silicone. (**h**) Scanning of the assembled phantom using the EinScan-SE 3D scanner (Shining 3D). (**i**) Digital model of the assembled phantom obtained with the scanner.

Then, the skull model was exported as a .stl file, and the open-source software Blender [42] was used to simplify the 3D model (i.e., by removing anatomical parts not involved in the simulation such as the orbital bones) and to add six semi-spherical markers (Ø 2 mm) on the skull surface simulating pre-operative implanted fiducial markers for registering the anatomy in the robot workspace. Markers were designed around the fractured portion (Figure 3a) to optimise the registration error at the surgical target.

#### 2.3.2. Phantom Design and Fabrication

The obtained digital model was edited to be fabricated using fused deposition modelling (FDM) by taking into account only half of the skull with the injured portion. This allowed an easier positioning on the print bed and a reduced amount of support (tree-like generated with Cura 5.0, Ultimaker, Utrecht, The Netherlands), as well as a shorter total print time (around 12 h). The model was 3D printed with the Creality Ender 5 3D printer using ivory poly-lactic acid (PLA) (Figure 3b,c). Bone fragments not completely broken (or fused together due to the segmentation) were manually separated by a neurosurgeon using a Dremel^®^, thus simulating the surgical procedure (Figure 3d,e). Based on the obtained skull phantom, the internal part representing soft tissues was designed (Figure 3f) and fabricated (Figure 3g) by casting Eco-Flex 00-10 silicone (E~20 kPa, similar to *dura mater* [43]) into a purposely designed mould. The two portions, bone and soft tissue were then glued using acrylic glue onto a poly(methyl-methacrylate) (PMMA) sheet to prevent relative sliding. The digital model of the assembled phantom was then obtained using a 3D scanner (EinScan-SE, Shining 3D) (Figure 3h). To improve the accuracy of the acquisition and prevent the presence of voids in the reconstructed mesh, the surface of the silicone was covered with talcum powder. In a real scenario, this step can be overcome since the digital model of the patient defect is directly obtained from the medical imaging step.

### 2.4. Printing Path Planning

#### 2.4.1. Non-Planar Slicing

**Figure 4:**
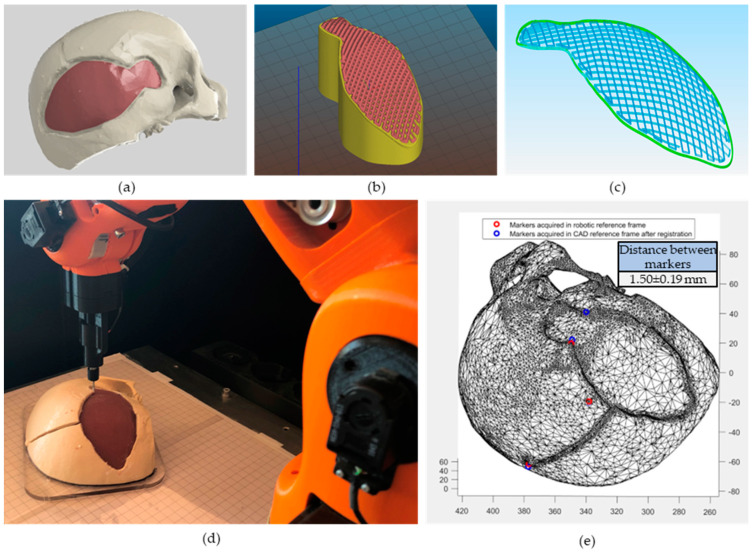
Printing path planning and registration. (**a**) Digital model of the scanned phantom: the red portion, corresponding to the damaged portion of the skull, was used for the path planning. (**b**) Non-planar slicing carried out in an experimental version of Slic3r. Non-planar layers are visible on the top of the imported model. (**c**) G-code of the top layers: the code exported from Slic3r was edited to remove the perimeter of the planar layers. (**d**) Acquisition of the fiducial markers in the IMAGObot reference frame: the touch probe is used to obtain the spatial coordinates of the markers. (**e**) Correspondence between the markers in the robot and CAD software reference frame after the registration procedure: RED = markers acquired in the robot reference frame, BLUE = markers acquired in the CAD reference frame after registration. Distance measured between markers after registration is 1.50 ± 0.19 mm.

The path planning for the in situ bioprinting step was carried out starting from the digital model acquired with the scanner (Figure 3i), which has been used as the pre-operative imaging modality. The assumption underlying the proposed approach is to consider a constant skull thickness at the defect—approximately 6 mm [44]. This allows the reconstruction of the damaged tissue through a 2.5D approach: the base layer (corresponding to the surface of the exposed *dura mater*) is repeated in height to reconstruct the missing bone tissue, thus filling the whole defect. The mesh file was uploaded on the CAD software Autodesk^®^ Fusion 360 and the defect portion was extracted (Figure 4a) to obtain the printing pattern. The .stl file of the defect volume was imported into an experimental version of the slicing software Slic3r [45], applying the non-planar slicing only to the top layer of the mesh (Figure 4b). Printing settings were the following: 50% top layer infill density, 0% planar layers infill density, 45° infill angle, 12 top layers, 0 bottom layers, 1 perimeter, 0.6 mm layer thickness. Since the Slic3r software does not allow selecting the infill density of the bottom and top layers, an extruder width twice (1.6 mm) as large as the needle actually used for the bioprinting step (0.8 mm) was selected to obtain a 50% infill density. The obtained g-code was exported and edited in Notepad++ to only extract the top layers’ path (Figure 4c) (the path corresponding to the perimeter of the planar layers was manually deleted). The z-coordinate of the obtained non-planar layers was adjusted following the registration procedure described in Section 2.4.2. Considering different applications where the damaged area to be reconstructed has not a constant thickness, a different approach could be followed: (i) the external surface (both top and bottom) can be fabricated using a non-planar slicing approach to ensure good adhesion on the substrate as well as good aesthetical and mechanical properties; (ii) the internal portion can be fabricated using a standard planar slicing.

#### 2.4.2. Registration Using Fiducial Markers

**Figure 5:**
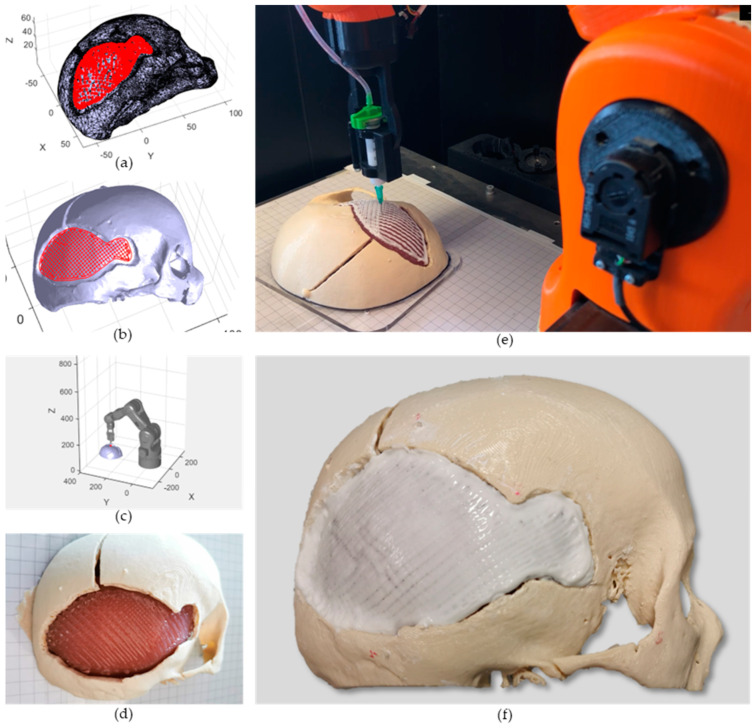
In situ bioprinting to regenerate the cranial defect. (**a**) Preview of the points of the printing pattern placed on the phantom mesh after the registration phase. (**b**) Preview of the printing pattern in the robot workspace: the porosity given by the 50% infill density is clearly visible. (**c**) Simulation of the end-effector movement following the non-planar approach in the IMAGObot Graphical User Interface. (**d**) Two layers of sacrificial support material (20% *w/v* Pluronic acid F127) bioprinted onto the *Dura Mater* to avoid direct contact with the bone phase. (**e**) In situ bioprinting step of the bone substitute with IMAGObot. (**f**) Final result of the cranial defect regeneration with the in situ bioprinting approach.

In order to obtain the most accurate registration, a point-based method has been employed on clearly distinguishable points. In particular, we selected on the phantom four fiducial markers, two artificial and two anatomical landmarks (consisting of vertex tips of the broken bones). Only two of the semi-spherical artificial markers on the phantom (Figure 3a) were available for registration as some of them were removed by the neurosurgeon with the movable bone fragments, while others were not clearly visible in the mesh reconstructed with the 3D scanner. The coordinates of the selected four points were acquired with the robot touch probe (Figure 4d) and identified in the reference frame of the CAD software (Figure 4e). As soon as the probe touches the desired points, the joint angles are extracted and saved in a .txt file. Using the Forward Kinematics algorithm, the spatial coordinates of these points in the robot reference frame are computed. The best-fitting rigid transformation that aligns the two sets of corresponding points was estimated via the least mean square method [46]. The registered printing pattern (Figure 5a) was then used for the calculation of IMAGObot joint angles using the inverse kinematic algorithm.

### 2.5. In Situ Bioprinting

Two layers of sacrificial support material (20% *w/v* Pluronic acid F127, Sigma Aldrich, Milan, Italy) were bioprinted onto the soft tissues in order not to deposit the bone substitute directly onto the *Dura mater*. This gives a two-fold advantage: (i) It allows a little gap to be left during printing between the bone tissue and the underlying tissue, enabling better cross-linking of the former without the risk of damaging the *dura mater*; (ii) it provides a homogeneous layer for the deposition of the upper layers, favouring their adhesion. The benefit of using pluronic acid is that it is easily removable after cross-linking the overlying tissue. The bone phase was then obtained through the deposition of ten layers of an osteoinductive hydrogel (solution of 50% *w*/*v* nanohydroxyapatite (nanoXIMHAp, Fluidinova, Maia, Portugal) in 10% *w*/*v* gelatin (Sigma-Aldrich, Italy) cross-linked with 0.2% *w*/*v* genipin (Challenge Bioproduct Co., LTD, Taichung, Taiwan) [47]). All the in situ bioprinting phases were carried out through pneumatic extrusion-based bioprinting: 0.5 bar and 0.7 bar pressures were used for sacrificial and bone substitute layers, respectively. The same printing parameters were used for both sacrificial and bone layers. An 18 G cylindrical needle and an average printing speed of 3 mm/s were used. The printability of the biomaterial ink was assessed with a predictive tool that we previously developed [48]. Briefly, the rheological and mechanical properties were obtained as described in [48] via rheological measurements using a HAAKE RheoStress 6000 rheometer equipped with a cone-plate (1° angle) measuring tool. Then, the obtained properties along with the print settings (maximum pressure: $1\times 10^{8}$ Pa; needle diameter: 0.8 mm; print speed: 3 mm/s) and scaffold dimensions (length, width: 50 mm; height: 6mm) were used as input for the online tool (https://rheo.herokuapp.com/form, accessed on 7 May 2023). As optimisation inputs, constrained layer height was set to 0.6 mm. Considering an in situ bioprinting procedure in vivo*,* the sterilisation of the biomaterial inks used should be taken into account. The treatment with ethylene oxide seems to be the most promising since it does not affect the biochemical and rheological properties of the hydrogels, thus not influencing the printability and osteo-inductive activity of the used materials [49].

**Figure 6:**
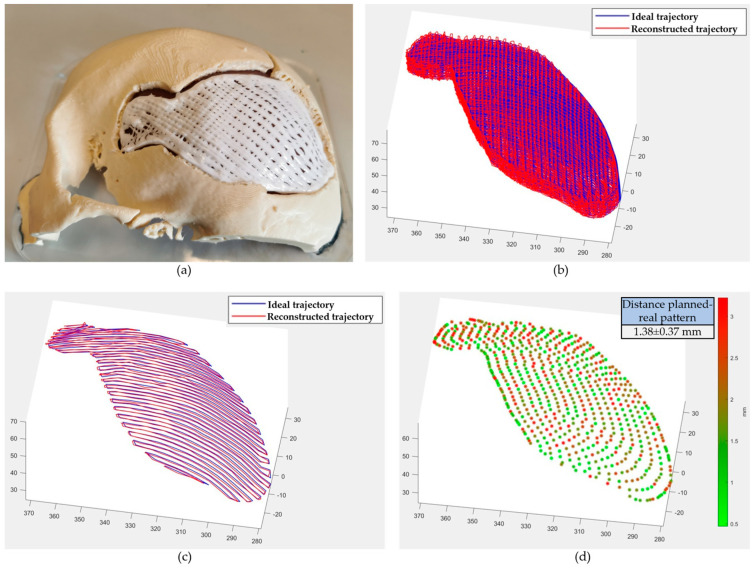
Quality of the cranial defect reconstruction. (**a**) Result of the cranial reconstruction during in situ bioprinting step (four layers printed). (**b**) Comparison between the whole planned and real (obtained from IMAGObot encoders) trajectory. (**c**) Comparison between a single planned printing layer and real trajectory. (**d**) Measured distance between the planned and real pattern. A single layer is displayed. Data on Cartesian axes are expressed in mm.

The quality of the printed pattern was quantitatively assessed by measuring the mean Euclidean distance between each point of the planned trajectory and the actually printed path. Data related to the real trajectory were acquired by storing joint angles values from LinuxCNC encoders variables in a .txt file. These values were obtained for each line of the g-code file during the in situ bioprinting step (Figure 6a). Using a Forward Kinematic algorithm, cartesian coordinates were obtained from the joint angles and the comparison with the planned pattern was performed in Matlab^®^.

## 3. Results and Discussion

### 3.1. Cranial Phantom Fabrication

The skull phantom was successfully 3D printed as can be seen in Figure 3b and the internal tree-like support was manually removed (Figure 3c). The internal part of the phantom (representing the *Dura mater*) was demoulded after the complete polymerisation of the Eco-flex 00-10 silicone (about 12 h) and the phantom was assembled as shown in Figure 3g.

### 3.2. Path Planning

Starting from the digital model of the segmented internal portion of the damaged area the non-planar path was computed using the *Slic3r* software (Figure 4b). The desired infill density of the top layers was obtained by setting the extruder width twice as the needle diameter actually used for the in situ bioprinting. This adjustment is acceptable as the slicer software is only used in this case to determine the coordinates of the trajectory points, ignoring the amount of material extruded for each segment of the pattern (strongly dependent on the extruder width), which was pneumatically controlled as discussed in Section 2.5. The g-code was cleaned of undesired paths (perimeter of the planar layers) and imported in Matlab^®^ for the registration step. We obtained a fiducial registration error of 1.50 mm (least mean square error, with a maximum error of 1.69 mm), using both anatomical and artificial markers. Given our experience, the availability of implanted artificial markers increases the possibility to find clearly distinguishable points to perform an accurate registration, but some of them can be removed during the initial steps of the intervention if not visible in the intra-operative scanner image. For this reason, a redundant number of artificial markers should be implanted.

The registered printing pattern was used to compute the joint angles in the IMAGObot app. The obtained pattern (Figure 5b) was simulated (Figure 5c) (see Supplementary Video S1) and then tested on the phantom.

### 3.3. In Situ Bioprinting

Following a standardised robotic-based in situ bioprinting workflow, IMAGObot was able to correctly restore the cranial defect through the localisation of the patient and the deposition of the biomaterials into the damaged area (see Supplementary Video S2). Two layers of Pluronic acid support were deposited onto the *Dura Mater* (Figure 5d) and, successively, 10 layers of the osteo-inductive hydrogel were bioprinted onto the sacrificial layers (Figure 5e,f). The ability of the structure to retain its shape was verified using the predictive tool described in Section 2.5. Biomaterial ink properties obtained from the rheological and mechanical characterisation are reported in Table 1. Briefly, with the desired printing settings and material properties, exploiting an infill density higher than 20%, a neglectable deformation of the entire structure is obtained.

The duration of whole printing procedure for the reconstruction of the 40 cm^2^ defect bone took about 1 h and 40 min. This is one of the main advantages of using the in situ bioprinting approach for the treatment of large cranial defects since the traditional surgical procedure, excluding the patient preparation time (e.g., anaesthesia, vital parameters monitoring) can take up to 3 h. Moreover, the additive manufacturing of metal cranial implants, mostly used in the current practice, can take up to 10–15 h, not considering the design phase. The timings of the various steps of the whole operational workflow and their accuracy are reported in Table 2. Even considering the entire workflow, the total time is still less than 2 h (excluding the imaging phase), which is an advantage over the traditional approach. This time would not vary considering a sterile robotic arm positioned in a real operating room. The time required for biomaterial ink sterilisation (>12 h) [49] is not considered in this work since it can be stored in sterile conditions in 5 mL syringes and used directly for the in situ bioprinting step. After the cross-linking of the bone phase (obtained after the printing phase), the Pluronic acid support was removed.

The printing quality was assessed by measuring the distance between the planned and the real trajectory. The two patterns were compared in Matlab^®^ (whole and single-layer printing paths are shown in Figure 6b,c). For each point, the Euclidean distance was measured and is shown in Figure 6d. The mean value resulted in 1.38 ± 0.37 mm, and due to the low mechanical rigidity of the robotic platform used compared to commercial systems it can be considered acceptable. This accuracy (reported in Table 2 as “in situ bioprinting”), even if far from the length scale of biological tissue organisation, compared to manual surgical instruments positioning represents an improvement and can thus reduce human error.

The approach of reconstructing the defect directly on the phantom mimicking the patient using a robotic arm gave very promising results, having the premises to become a valid alternative to the current procedure based on the surgeon’s expertise (greater precision during the intervention and shorter duration). In addition, the biomaterial used for bone regeneration would promote better integration with the residual bone, thus leading to a faster recovery by the patient. In fact, as reported by Chiesa et al. [47], the use of a gelatin-nanohydroxyapatite structure with interconnected porosity (given by the 50% infill percentage and the alternation in the fibre direction of two successive layers) promotes cell adhesion and migration, thus favouring osteogenesis. A biological characterisation over time will be further carried out to assess the behaviour of the printed construct in the physiological environment and its integration with the host tissue. Furthermore, considering current surgical practice, in the case of a large cranial defect a replacement plate has to be fabricated and anchored to the residual bone. Such plates can lead to complications for the patient, including pressure ulcers, and a further cranioplasty is required in these cases [50]. Owing to in situ bioprinting, this step could be overcome by printing the replacement tissue directly in the damaged area. Even considering the thickness of the substitute tissue, in fact, the promotion of the internal vascularisation favoured by the used biomaterials may decrease the limitations of the scaffold-based approach. Moreover, the use of a printing system based on pneumatic extrusion also allows the use of a wide range of biomaterials and is therefore suitable for the treatment of various and heterogeneous biological tissues (e.g., defects involving more than one tissue, such as bone, cartilage, skin). In this regard, the use of an in situ bioprinting approach represents an additional advantage over conventional implant-based techniques, since the mechanical properties of the biomaterial inks used are much closer to that of the native bone tissue, thus not leading to a stress shielding effect. Furthermore, the cost of the entire procedure would be reduced since the use of expensive equipment as EBM systems (>$1 million) will not be needed.

Finally, comparing the workflow proposed in this study with that proposed by Fortunato et al. [33], the registration step is now required. In the previous study, in fact, it was not necessary since it was the robot itself that was used to obtain the defect digital model. The workflow proposed in our work can instead be applied for any in situ bioprinting approach where the digital model of the damaged area is acquired with an instrument external to the robot (e.g., CT), and a registration/localisation step is thus required. Moreover, the versatility of the IMAGObot platform, due to a fast and easy tool-change, allows the use of multiple printheads (e.g., extrusion-based, valve-jet, ink-jet) and thus the fabrication of multi-scale and multi-material structures, favouring the regeneration of also more complex biological tissues.

## 4. Conclusions

In this study, we presented the application of in situ bioprinting to a simulated clinical case study. This technology was used to simulate the restoring of the bone in an injured head, showing a standardised robotic-based workflow onto a purposely designed phantom. The successful regeneration of the cranial defect, in terms of damaged area filling, proved the great potential that the robotic approach can have for in situ bioprinting applications in cranial surgery. Furthermore, looking at the whole procedure, this approach is also promising for more advanced clinical applications, such as remote robotic surgery, where the surgeon can acquire data about the patient and, after programming the operating phase, remotely control the robot to regenerate the damaged site. Future investigations will be focused on the testing of the proposed approach both in vitro and in vivo, to analyse the osteo-integration of the in situ bioprinted implant with the native tissue.

## Figures and Tables

**Table 1:** Gelatin+nanohydroxyapatite biomaterial ink rheological and mechanical properties.

Rheological Properties	Mechanical Properties
Herschel-Bulkley model	$\mathrm{Elastic}\;\mathrm{modulus}:\;5\times 10^{4}$ Pa
Flow index range (n): 0.02	$\mathrm{Zero}\text{-}\mathrm{shear}\;\mathrm{viscosity}:\;5\times 10^{4}$ Pa·s
Consistency factor: 10 Pa·s^n^	Poisson modulus in range: 0.49
Yield stress: 500 Pa	Density: 1000 kg/m^3^

**Table 2:** Duration and accuracy of all the steps of the in situ bioprinting workflow.

	Duration	Accuracy
Preparation of the surgical area (removal of bone fragments)	15 min	N.A.
Acquisition of the 3D model (Computed Tomography)	5 min	0.4 mm
Path planning	10 min	<0.1 mm
Registration	5 min	~1.5 mm
In situ bioprinting	1 h 40 min	~1.5 mm

## Data Availability

Data will be made available on request.

## Supplementary Materials

The following supporting information can be downloaded at: https://www.mdpi.com/article/10.3390/bioengineering10050560/s1, Video S1: Supplementary_video1.mp4; Video S2: Supplementary_video2.mp4.
